# In Situ Real-Time Quantitative Determination in Electrochemical Nuclear Magnetic Resonance Spectroscopy

**DOI:** 10.3390/s22010282

**Published:** 2021-12-31

**Authors:** Min Liu, Zu-Rong Ni, Hui-Jun Sun, Shuo-Hui Cao, Zhong Chen

**Affiliations:** Department of Electronic Science, Fujian Provincial Key Laboratory of Plasma and Magnetic Resonance, State Key Laboratory of Physical Chemistry of Solid Surfaces, Xiamen University, Xiamen 361005, China; liumin@stu.xmu.edu.cn (M.L.); sunhj@xmu.edu.cn (H.-J.S.); shuohuicao@xmu.edu.cn (S.-H.C.); chenz@xmu.edu.cn (Z.C.)

**Keywords:** qNMR, electrolyte, electrochemical, external standard, EAOPs

## Abstract

For the purpose of acquiring highly sensitive and differential spectra in in situ electrochemical nuclear magnetic resonance (EC-NMR) spectroscopy, uniform distributions of amplitudes and phases of radio frequency (RF) fields in the sample are needed for consistent flip angles of all nuclei under scrutiny. However, intrinsic electromagnetic incompatibility exists between such requirements with electric properties of the conductive material in an electrolytic cell, including metallic electrodes and ionic electrolytes. This proposed work presents the adverse repercussions of gradually varying electrolyte conductivity, which is strongly associated with the change of ion concentrations in a real-time electrochemical reaction, on spatial distributions of RF field amplitude and phase in the detective zone of an NMR probe coil. To compensate for such a non-linear trend of the spatial dependent distribution, we eliminate different excitation effects of the RF field on the build-in external standard and the electrolyte both situated in nearly the same detection area, as well as promote the greater accuracy of quantitative determination of reactant concentrations. The reliability and effectiveness of the improved in situ EC-qNMR (quantitative NMR) method are confirmed by the real-time monitoring of the electrochemical advanced oxidation process for phenol, in which instant concentrations of reactants and products are detected simultaneously to verify the degradation reaction scheme of phenol.

## 1. Introduction

Nuclear magnetic resonance (NMR) spectroscopy qualifies as one of the most versatile non-invasive methods for structural elucidations of synthesized compounds, natural products and biomolecules [1,2,3]. Understandably, the information obtained from NMR spectra is related to quantitative analyses [4,5,6]. As a standard-free quantification method, quantitative NMR (qNMR) secures its own unique advantages in comparison with traditional quantitative determination tools, such as the chromatographic spectroscopy hyphenated technology. Due to the direct proportionality between integrated intensities of a resolved signal and the number of nuclear spins, qNMR is generally conducive to quantification of entire components in multiple compound mixtures and does not require chemical identical standards. In addition, because high-resolution NMR spectra depends sensitively on minor chemical structural changes, the component comprising 0.1% or less of the compound mass can be detected quantitatively when a carefully designed measurement procedure is implemented [7].

For pure electrochemical behaviors, active electrode potential controls are employed, by which molecular level relationships between electronic/geometric structures with dynamic reactivity can be obtained uniquely as a function of electrode potential at the nanoscale, particularly under the same (or close) conditions as (to) those in real-world practical applications. Benefitting from those merits mentioned above, integrating high-sensitivity NMR spectroscopy with electrochemistry (EC) promises direct observation of electrochemical products, especially unstable intermediate ones, in an in situ electrolytic cell [8,9,10,11,12,13]. Another superiority of in situ EC-qNMR is demonstrated by tracking quantitatively the behavior of specific marker atoms in the reaction, rendering itself the most effective tool to explore reactant-activation processes and catalytic-reaction mechanisms at the molecular level [14,15,16].

Despite these unique merits of EC-qNMR, some technical shortcomings of electromagnetic incompatibility, which arise from the combination of the electrochemistry setup and the NMR spectrometer [17,18,19], persist inevitably. In particular, both the presence of different materials with incompatible magnetic permeability in an electrochemical setup and the presence of the electrolysis current conducting through the electrodes and electrolyte account for a disturbance of the NMR static magnetic field *B*_0_, and then inevitably decrease the spectral resolution [20,21,22]. Based on Faraday’s Law, eddy currents are induced by the time-varying radio field (RF) in the coil of an NMR spectrometer and create an auxiliary magnetic field relevant to the conductivity of metal electrolytic electrode and conductive electrolyte, which normally includes soluble salts, acids, or bases. Also, regardless of the initial applied uniform field, the total magnetic field consisting of the externally applied and induced magnetic field tends to deviate from homogeneous field distributions.

Consequently, such repercussions on the uniform distribution of the amplitude and phase of the RF field even inside a homogeneous sample lead to an uneven spatial response in NMR and worsen as the electrolyte conductivity changes, which is attributed to the consecutive variation of ion concentration in electrochemical reaction. Furthermore, due to the ohmic loss of eddy currents flowing in the electrochemical electrode and electrolyte, the RF power dissipation in EC-qNMR cell deteriorates the NMR signal to noise ratios (SNRs) at the same time. Critically, all these factors reduce the optimal performance of the NMR technique in an electrochemical analysis and prohibit the quantitative determination of reactants from reaching high accuracies. Unfortunately, to the best of our knowledge, papers aiming to remedy these aforementioned shortcomings have rarely been reported.

This paper presents an EC-qNMR electrolytic cell, accommodating coaxial insertion capillary and external reference standards. The uniformity of the RF field amplitude and phase inside the sensitive volume of the NMR probe is analyzed theoretically to evaluate the influence of the electrolyte with different electrical conductivity, and such a negative impact on the quantitative determination of the analyte is revealed by in situ EC-qNMR experiments. A corresponding calibration method is utilized to compensate the distortion of the RF field in the electrochemical reactant and standard so that the accuracy of quantitative determination can be improved. The method is validated by the study of the proposal reaction route for phenol in the electrochemical oxidation degradation process.

## 2. Experimental

### 2.1. Reagents and Instruments

All chemicals used for electrolysis are reagent grade and included ethylene glycol (99%), isopropyl alcohol (99.7%), benzene (95%) and phenol (99%). High-quality water (Millipore Milli-Q, resistivity > 18 MΩ·cm) was employed for the preparation of all solutions. All chemicals for nanoparticles preparation (HAuCl_4_, NaBH_4_, sodium citrate, 3-aminopropyltrimethoxysilane (APTMS, 97%)) were purchased from Sigma–Aldrich Inc., Shanghai, China. In NMR studies, deuterium oxide (99.8% D, Aldrich) was used to prepare deuterated solutions. ^1^H NMR spectra were acquired with a Varian 500 MHz spectrometer and the chemical shifts were aligned with reference to the signal of TMS. According to the test sample with low concentrations (<50 mM), the number of acquisition was 32 times and each acquisition time was kept no greater than 3 min.

Electrochemical measurements were carried out in a lab-made EC-qNMR cell with a computer-controlled Labnet UI5502 potentiostat, of which the sweep rate was maintained at 50 mV/s unless otherwise stated. A Pt plate and Ag/AgCl wire electrode (SCE) were used as counter and reference electrodes, respectively. All potentials are referred to the Ag/AgCl electrode in this paper. The working electrode was constructed from thin 50 nm palisade gold film (PGF) deposited on the outer wall of a 2 mm-o.d. (outer diameter) capillary. Prior to an EC measurement, the PGF electrode was processed by the cyclic voltammetry (CV) cleaning in a 0.1 M H_2_SO_4_. The solution was also deaerated by ultrapure nitrogen for 15 min prior to any measurements, and its conductivity was measured with a DDS-307 (Yueping, Shanghai) conductivity meter.

### 2.2. Electrochemical Quantitative Nuclear Magnetic Resonance (EC-qNMR) Cell with Palisade Gold Film (PGF)

**Figure 1:**
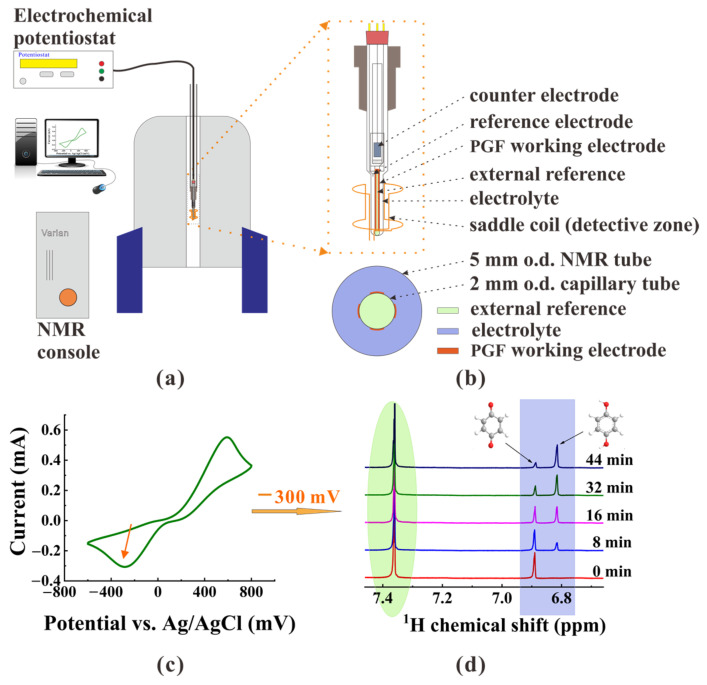
(**a**) Schematic of an in situ electrochemical quantitative nuclear magnetic resonance (EC-qNMR) setup. (**b**) Sectional view of the EC-qNMR cell, wherein the NMR effective detection zone. (**c**) Cyclic voltammogram of p-benzoquinone with a palisade gold film (PGF) electrode system in an acid solution. (**d**) In situ NMR spectra of the redox of p-benzoquinone at a potential of −300 mV, where the colors of the spectrum marker correspond to the compound in subgraph (**b**).

To increase the RF field homogeneity in the volume of substances, we presented an original EC-qNMR cell with palisade structure of working electrode that was suited for routine operations in a modern high-frequency NMR spectrometer [23], as shown in Figure 1. The homemade electrolytic cell was fabricated from several glass tubes with different outer diameters. A half-open capillary tube with outer surface deposited by the thin gold PGF served as working electrode. The counter electrode included a cylindrical black platinum plate and was placed inside another tube, which was then sealed by porous glass frit, preventing any byproducts generated at the auxiliary electrode from contaminating the main test solution at the working electrode. A 1 mm-o.d. Ag/AgCl wire served as the reference electrode, and its end was located as near the working electrode as possible for higher measurement precisions of the electrical potential. An external reference standard adapted to the reaction compound was poured into the half-open capillary tube, which was then airproofed via a Teflon band. Therefore, due to the comparatively unaffected concentration of isolated external reference, the concentration variation of electrochemical reactants can be calibrated conveniently in real-time.

## 3. Results and Discussion

### 3.1. Intrinsic Effect on the NMR Spectra by the Samples with Different Conductivity

Quantitative determination of NMR is based on the most important fundamental relationship between numbers *N* of protons at relevant functional groups and corresponding spectral peak areas *I*, as shown in Equation (1) below,

(1) I=K⋅N

where *K* denoted the instrumental factor, which is related to the static magnetic field *B*_0_, applied external RF field *B*_1_ and environment temperature *T*. Consequently, the absolute amount or the concentrations of the mixture components are usually determined indirectly by the relative ratio of each peak area, when a structurally distinct reference is utilized as the internal or external standard. Even under this treatment, both approaches are not suited for the EC-qNMR technique. First, finding an appropriate internal standard can constitute a time-consuming task when both the chemical reaction with the component (even impurities) or the solvent and the overlap in the complicated spectrum of samples are avoided simultaneously. Second, an external standard substance with known purity is prepared as a reference solution and is placed in a separate NMR tube with identical precision for NMR data acquisitions to ensure that both NMR spectra are acquired in a similar experimental condition (same *K* value in Equation (1)). However, any variations generated from different experimental conditions, after the electrochemical reaction begins, can affect the accuracy of the quantitative determination. As a result, this method cannot be properly adopted in EC-qNMR.

Alternatively, combining roles of internal and external standards in the traditional qNMR method, a homemade EC-qNMR cell includes a specialized NMR tube with extra co-coaxial capillary, into which the reference with a known purity is poured not only to avoid contamination of the analyte or interference of the chemical reaction, but to be further used for quantitative analysis. The absolute amount of sample is obtained by comparing the relative peak area of specified group proton of the external standard with that of analyte so that the concentration of the electrolyte can be expressed in Equation (2) as:

(2) $$C_{x}=K^{\prime }\cdot \frac{I_{x}}{I_{s}}\cdot \frac{N_{s}}{N_{x}}\cdot \frac{V_{s}}{V_{x}}\cdot C_{s}$$

where *C_x_* and *C_s_* are concentrations of the sample and external standard, *V_x_* and vs. are sample volumes in the tube and capillary, both of which are located in the effective detection zone of the NMR spectrometer probe, respectively. The ratio of these two volumes should be pre-calibrated by adding known purity internal standard into the sample, as shown in Equation (3),

(3) $$\frac{V_{s}}{V_{x}}=\frac{I_{s}}{I_{Is}}\cdot \frac{N_{Is}}{N_{s}}\cdot \frac{C_{Is}}{C_{s}}$$

where the subscript *I_s_* represents internal standard. For the purpose of avoiding the interaction from the internal standard in real operation, the ratio can be provided by the initial concentration of the reagent.

Coefficient *K*′ in Equation (2) represents different effects produced by the magnetic field in which the external standard and analyte are placed, including static magnetic field *B*_0_ and RF field *B*_1_ when RF irradiation and detection are performed by the same NMR probe coil. Despite the fact that the homogeneity of static magnetic field *B*_0_ may be worsened by those components with different magnetic susceptibility in an EC-qNMR cell, such adverse effects caused by the inconsistency of material properties can be removed practically by the cell’s specialized structural design. For instance, if the long lengths of those electrolysis chambers are parallel to the static magnetic field *B*_0_, nearly no changes in the resolution of NMR spectrum, which are acquired after the careful shimming of the field, suggest that the heterogeneity of the static magnetic field can be ignored. Therefore, since the shift of temperature *T* can also be kept null conveniently in a modern NMR spectrometer, the main factor affecting coefficient *K*′ is derived from the applied RF field ***B***_1_ due to the disturbing of conductive solution.

Based on the reciprocity principle first introduced by Hoult and Richards [24,25], the impact of the applied RF field on the intensity of NMR spectrum of the electrolyte and external standard can be evaluated quantitatively. In other words, the electromotive force (EMF) induced in a single turn of an axially symmetric loop by the time-dependent magnetic moment vector ***M*** of NMR spin precession at a specific point of the sample should act proportionally to the strength of the magnetic field vector ${\hat{B}}_{1}$ in the same position produced by per unit current flowing in the loop. Therefore, the total EMF *ξ* sums up contributions from nuclear moments across the whole sample, as described in Equation (4), i.e.,

(4) $$\xi =-\int_{v}{\hat{B}}_{1}\cdot \frac{\partial M}{\partial t}dv$$

As the sample size becomes comparable with the wavelength at high frequency, especially in a conductive aqueous solution, these conduction and displacement currents flowing in the sample generate their own fields with different phases, diversifying the amplitude and the phase of magnetic field ***B***_1_ from point to point in the conductive sample (see the analytical derivation in the Supplementary Materials SI). Consequently, the distortion of the amplitude and phase involved in the NMR RF field of different conductive samples tends to be non-linearly intensified as the conductivity of the samples varies instantaneously.

Besides this trend, the highly-conductive electrolyte alleviates the quality factor *Q* value of the NMR probe. Since the intensity of the NMR signal is proportional to the *Q* value, the reduction of *Q* value implies the decrease of both the signal strength and the signal noise ratio (SNR) in EC-qNMR experiments. On the other hand, the lowered *Q* value also increases the width of the 90° and 180° RF pulses, which can be more conveniently acquired for the detection and correction of damping *Q* value. Hence, the inverse relation between the width of pulse and the NMR signal intensity plays a key role in the conventional calibrated method when both NMR signals of the sample and separate external standard are driven and acquired by the same probe which is carefully tuned to match the consistency of experimental conditions [26,27]. However, in an in situ EC-qNMR experiment, the change of electrolyte conductivity indicates the variation of impedance load in a routine NMR probe coil, and the mechanical tuning and matching should be adapted continually during the electrochemical reaction. Generally, and practically, the tuning of the NMR probe is properly implemented in the preparing period before the electrochemical reaction starts. Namely, the width of the 90° or 180° RF pulse cannot be conveniently optimized in a real-time fashion. Thus, the constant width the 90° pulse throughout the entire reaction time actually disqualifies the conventional calibration method from correcting the intensity of reactants in the EC-qNMR experiments.

### 3.2. Choice of the External Standard and Its Concentration in EC-qNMR

To ensure the accuracy and reliability of the determination in EC-qNMR, an external standard with the same radicals as the reactant may be the first choice, since the nearly similar relaxation times of ^1^H nuclei in a consistent chemical environment make nuclear spin magnetizations restore thermal equilibrium almost at the same time, which promises that the integral area represents the whole contribution of all ^1^H nuclei. On the other hand, the quantitative characteristic peaks of the external standard and reactant with the same radicals usually appear closely, and can be easily interfered with those of intermediates and products. Therefore, the selection of an appropriate external standard that can be separated and easily identified in the EC-qNMR would be rather cumbersome and require multiple attempts.

The potential for errors of concentration determination increases when the striking discrepancy between integrated areas of characteristic peaks of the electrochemical reactant and standard exists. Consequently, the concentration of the external standard should be selected appropriately to make its peak areas comparable to or slightly less than that of the initial reactants. Furthermore, the careful post-processing of the NMR spectrum, including phase adjustment and baseline correction, is crucial to maintaining higher accuracy, especially in the determination of intermediates and products with low concentrations.

### 3.3. Method of Calibration in EC-qNMR

Before achieving the accurate quantitative analysis of signal strengths of the built-in external standard and analytes, a calibration method should be adopted to suppress negative effects caused by varying conductivities of electrolytes in the EC-qNMR cell. Analytes determined quantitatively for the calibration method are known as ethylene glycol (0.5 M) and isopropanol (0.1 M), both of which are prepared gravimetrically and dissolved in D_2_O solutions, respectively. Then the former was filled into a capillary tube alone as an external standard, and the latter was mixed with sulfuric acid at different concentrations (0.1–2 M) to imitate the electrolyte with different conductivities.

**Figure 2:**
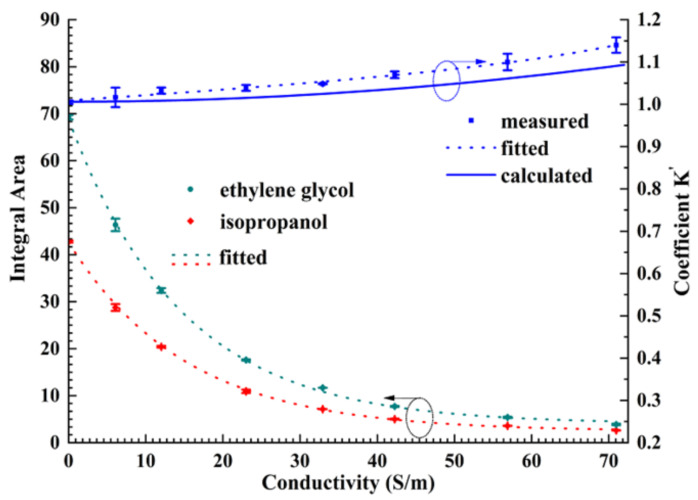
Relative ratio of the signal strength of built-in external standard and the sample with different conductivity, which corresponds to the trend of coefficient *K*′.

Clearly, both integral areas of spectra of the electrolyte and external standard decay monotonically as the conductivity of the electrolyte increases in the EC-qNMR cell (Figure 2). According to Equation (3), when the conductivity of the electrolyte solution approaches zero, the relative ratio of volume approaches 5.06, which coincides closely with the volume ratio 5.10 calculated roughly from the nominal dimensions of 5 mm-o.d. tubes. Furthermore, as the conductivity of electrolyte rises, the increase of measured coefficient *K*′ meets the trend of calculated *K*′, representing different effects on the external standard and the analyte by the applied alternative RF field *B*_1_. While the conductivity of the electrolyte approaches 20 S/m, coefficient *K*′ grows nearly 5%, indicating that the alternative RF field *B*_1_ is excluded from the center zone of the coaxial capillary gradually. The difference between calculated and measured coefficients *K*′ may arise from oversimplified models of the conductive solution and the NMR probe coil, in both of which the applied alternative RF field is treated as a homogeneous field and the non-uniform field at the edge of the sample is neglected in the theoretical analysis.

Since the concentration of the built-in external standard is maintained at a constant value during the electrochemical reaction, changes in the intensity of the standard reflect substantially the variation of the conductivity of the electrolyte. Consequently, based on the fitted curves as shown in Figure 2, the difference in the conductivity relative to its original value recorded at the beginning of the experiment can be derived from the variation of the integral area of the external standard from its initial value as well as the coefficient *K*′. Furthermore, in an in situ EC-qNMR experiment, even if the external standard may be different from the chemical compound (ethylene glycol here) used in the calibration procedure, the fitted curves, which only represent the influence of the electrolyte’s conductivity, are still applicable provided that variations in integral areas of external standards are first normalized to their initial values. Now, the initial integral area of the external standard in Figure 2 corresponds to the conductivity of the analyte acquired at the beginning of the EC-qNMR experiment. Taking into account the coefficient *K*′ correction, the improvement in the linearity of concentration can be found in the Supplementary Materials SII.

### 3.4. EC-qNMR Performance: Advanced Electrochemical Oxidation Process for Phenol Degradation

In recent years, electrochemical advanced oxidation processes (EAOPs) have been developed for the purpose of eliminating the environmental pollution with the focus on water treatments [28,29,30,31]. Herein, we investigate the EAOPs for the degradation mechanism of phenol to verify the reliability and effectiveness of the improved in situ EC-qNMR method and propose its degradation pathway.

**Figure 3:**
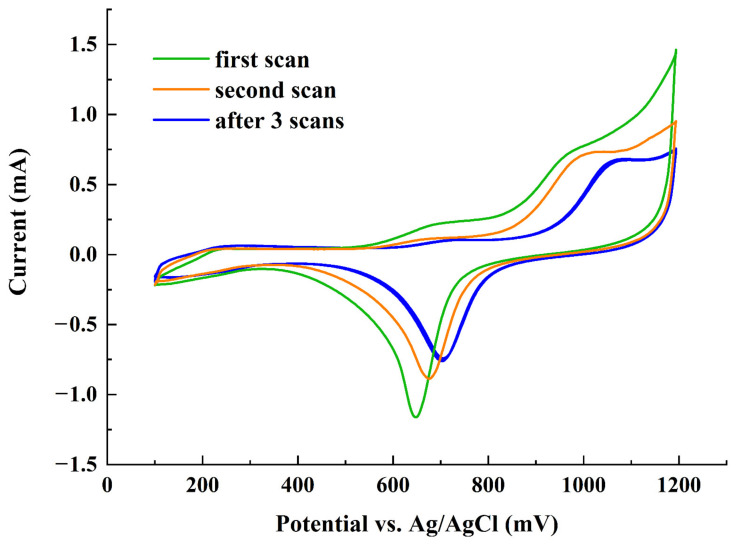
In situ cyclic voltammograms of PGF Au in 10 mM phenol + 0.1 M H_2_SO_4_ at 50 mV/s. Potentials were quoted against the Ag/AgCl.

The 10 mM phenol in 0.1 M H_2_SO_4_ is treated inside the EC-qNMR cell, and its in situ cyclic voltammograms are illustrated in Figure 3. Although the anodic peak current decreases slightly in first two consecutive cyclic voltammetry scans, no decrease of the anodic peak current is observed after the third scan, indicating that the electrode can operate steadily during the electrocatalytic degradation of phenol.

**Figure 4:**
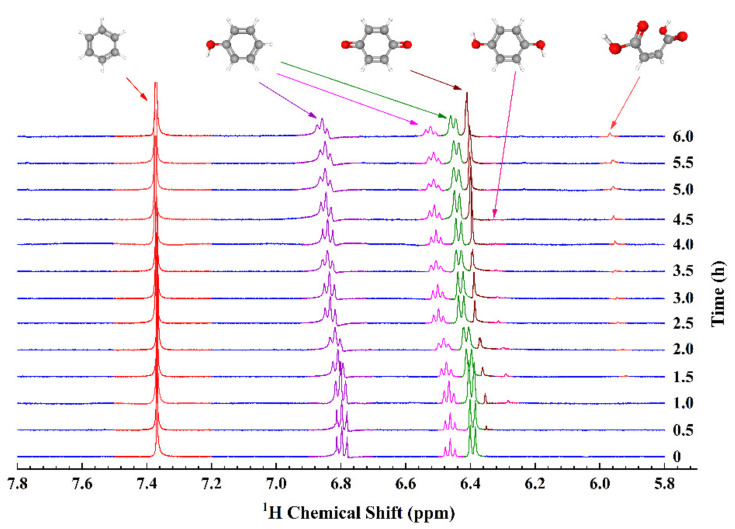
Time resolved in situ ^1^H EC-qNMR study of EAOPs in 10 mM phenol + 0.1 M H_2_SO_4_ at +1.1 V (vs. Ag/AgCl), the external standard is 25 mM benzene + DMSO.

Subsequently, a series of in situ ^1^H NMR spectra are acquired in equal time intervals as the working electrode is held at +1.1 V with respect to the Ag/AgCl quasi-reference electrode (Figure 4). Singlet peaks at 7.37 ppm, 6.41 ppm, 6.33 ppm, and 5.96 ppm correspond to the standard sample benzene (BZ), the reaction inter-mediate products p-benzoquinone (PBQ), hydroquinone (HQ), and maleic acid respectively, and the remaining three sets of multiple peaks (6.80 ppm, 6.46 ppm, and 6.39 ppm) are assigned to the reactant phenol. Owing to the consumption of phenol, its intensity continues to decrease, whereas the peak of PBQ increases steadily in the electrolysis reaction progress. Half an hour after the reaction has started, an HQ peak singleton appears, but nearly diminishes entirely 3 h later. During the period of degradation reaction, a faint single peak of maleic acid becomes conspicuous gradually. Another noteworthy phenomenon, in which each reactant peak moves slightly towards the low field (high frequency) direction besides the standard sample BZ, takes place in the reaction progress. It is exemplified by the shift of the multiplet center of phenol from an original 6.80 ppm to 6.85 ppm. The reason for these shifts lies in that the solution pH value decreases as the degradation proceeds. This reduction is evidenced by the variation of electrolyte conductivity detected prior to and after the experiment, from 4.3 S/m to 8.4 S/m. Nonetheless, the standard benzene is isolated from the electrolyte, leading to an unchanging chemical shift.

**Figure 5:**
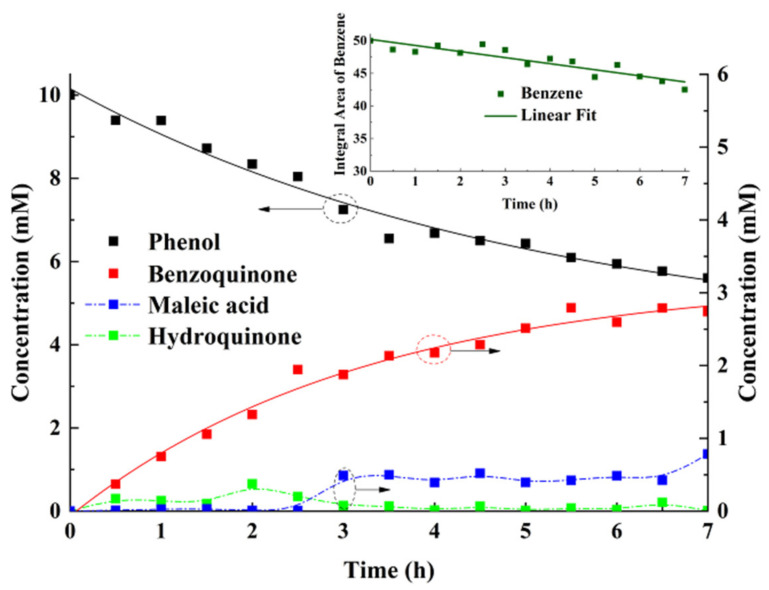
Evolution of concentrations of the reagent and products during the EAOPs for phenol in an in situ EC-qNMR cell. The reagent is phenol. The products are benzoquinone, hydroquinone, and maleic acid. Inset shows the integral area of benzene (standard sample) and its linear fitting line.

Because of the slight environmental instability and the non-long-term adaptability in the experimental procedure and setup, the integrated area of the standard sample BZ varies all the time (Figure 5). Nevertheless, perceived by the linear fitting curve (the green straight line in the inset), its slow decreasing trend against time indicates the inevitable effect primarily exerted by variation of solution conductivity on the BZ intensity. Hence, the standard benzene behaves equivalently as an in situ probe that can be used to monitor slight changes in the physical and chemical environment, in which the sample is immersed. Accordingly, based on the conductivity 4.3 S/m of the analyte measured at the beginning of the experiment, the integrated area of BZ is normalized by the corresponding standard integrated area 50.0 regarding the inset in Figure 5.

These normalized values at different sampling moments indicate the real-time conductivity of analytes, as shown in Figure 2. Consequently, the corresponding correction factor *K*′ updates the concentrations of different species separately in the reaction process, as magnified in Figure 5.

The phenol concentration decreases exponentially from 10 mM to 5.6 mM after 7 h, indicating a nearly 44% consumption rate. The BQ concentration steadily rises to 2.8 mM, whereas the pre-reactant HQ concentration reaches a maximum of 0.48 mM at 2 h and then fluctuates within a small range close to zero for the rest of the time. In order to conduct the kinetic analysis of phenol degradation in the EC-qNMR, rate constants for the formation or consumption of species are estimated, supposing they follow evolutions comparable to first-order reactions. The phenol is first degraded to reaction intermediates with an overall kinetics constant of 0.092 h^−1^. In most cases, the phenol adsorbed on a planar gold electrode surface loses electrons and is then oxidized into products such as HQ, PBQ, and other phenolic radical intermediates. Then, insoluble polymers arising from the interaction of these intermediate products or themselves adhere to the surface of the working electrode and prevent the reaction from proceeding. Despite the limited decomposition rate of phenol, the metallic nanoparticles electrode retains strong electrocatalytic and antifouling capability as a result of the increased electrode porosity and surface. Primarily due to no detection of catechol in the investigation, the decomposition of phenol into HQ, which is then oxidized to PBQ, can be presumed to be two consecutive first-order reactions. Furthermore, since carbon dioxide cannot be quantified using EC-qNMR, only the lower limit of the PBQ formation rate, evaluated by ignoring the amount of PBQ converted to organic acids, is 3.1 h^−1^. Obviously, the formation of HQ is the rate-determining step in the generation of PBQ.

**Figure 6:**
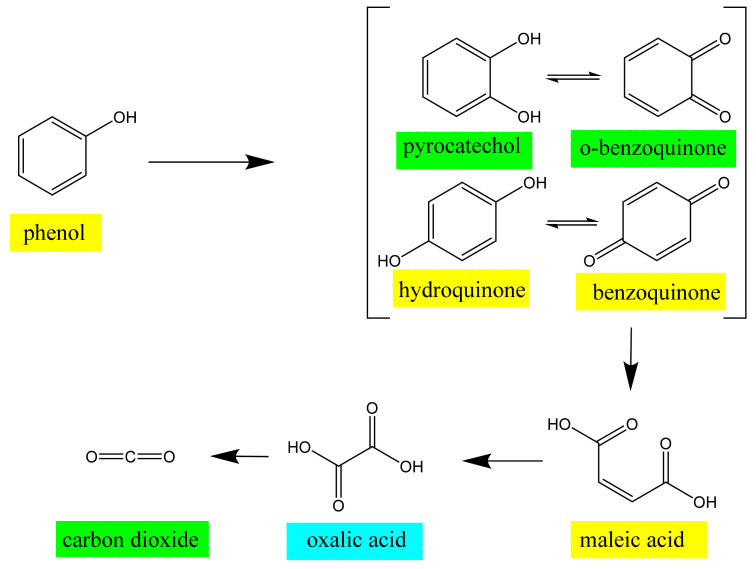
Mechanism of electrochemical oxidation degradation process of phenol, proposed on the basis of our NMR data and previous reports [32,33]. The chemical species highlighted in yellow were observed in this study, those highlighted in cyan was validated in an ex situ EC experiment, and those highlighted in green in previous studies using other analytical techniques.

Accordingly, Figure 6 presents a general initial reaction sequence proposed from the organic intermediates perceived by in situ EC-qNMR mentioned previously. In the initial stage of EAOPs for phenol, the phenol, H_2_O, and OH^−^ in the solution diffuse and are adsorbed onto the surface of the anode. Continuously, they bring electrons to the electrode, generating reactive intermediates. The former generates phenolic oxygen radicals, and the latter two generate hydroxyl radicals (-OH) individually. Phenolic oxygen radicals adsorbed on the surface of the electrode undergo an electron transfer reaction, in which they are oxidized first to HQ and then to PBQ. Intermediate hydroxyl radicals that diffuse into the solution also oxidize phenol into HQ, which is oxidized to PBQ on the electrode surface subsequently. Although final products of these two reaction pathways all become PBQ, the fast reaction of oxidizing HQ into PBQ contributes further to the low concentration of HQ in the solution. In addition, primarily due to the low concentration of trace amounts of catechol, PBQ prevails in the reaction system as the main intermediate product. As the reaction progresses and the concentration of the phenol decreases continually, water molecules dominate the competitive adsorptions on the electrode surface over hydroxide ions, prompting the generation of abundant hydroxyl radicals. Consequently, highly active hydroxyl radicals oxidize the organic matter, producing some small molecules of organic acids, such as maleic acid. Finally, the carbon chain is broken to form oxalic acid, the existence of which is verified by an ex situ EC-qNMR experiment. Calcium sulfate is added to the electrolyte to monitor the electrochemical degradation of phenol. The white deposits were observed in the solution, indicating that oxalic acid was generated and precipitated out by Ca^2+^. The oxalic acid is further oxidized into CO_2_ and H_2_O at the end. However, those intermediate product quinones eventually exist, indicating that a small part of electrochemical conversion continues to occur.

## 4. Conclusions

We report here the effects of the varying conductivity of the electrolyte, which is present in an electrolytic cell with coaxial insertion capillary containing a built-in external reference standard, on both the spatial dispersion of the amplitude and the phase of the RF field. In order to improve the precision of the quantitative determination of the substances, methods are performed to calibrate the NMR signal integration intensity of both external standard and the substance under test to compensate for the highly non-linear trend of the spatial distribution of the varying amplitude and phase of the RF field, as the conductivity changed in real time during the electrochemical reaction in EC-qNMR. Finally, the verification of the design and approach is achieved by quantitative determination of the reactants in EAOPs for phenol and validation the scheme of phenol degradation.

## Data Availability

Data is contained within the article or Supplementary Materials.

## Supplementary Materials

The following supporting information can be downloaded at: https://www.mdpi.com/article/10.3390/s22010282/s1, SI: Skin effect of the hollow cylindrical conductive electrolyte; SII: Linear regressions of the NMR results versus gravimetric concentration.
