# Supplementary material for: In Situ Real-Time Quantitative Determination in Electrochemical Nuclear Magnetic Resonance Spectroscopy
Source: Sensors (Basel). 2021 Dec 31;22(1):282. doi: 10.3390/s22010282 (PMC8749650; doi:10.3390/s22010282)
Supplement: Supplementary file 1 [file sensors-22-00282-s001.zip › sensors-1490728-supplementary.pdf]

Supplementary Materials

In situ Real-time Quantitative Determination in Electrochemical Nuclear Magnetic Resonance Spectroscopy

Min Liu, Zu-Rong Ni *, Hui-Jun Sun, Shuo-Hui Cao and Zhong Chen

Department of Electronic Science, Fujian Provincial Key Laboratory of Plasma and Magnetic Resonance, State Key Laboratory of Physical Chemistry of Solid Surfaces, Xiamen University, Xiamen, China; liumin@stu.xmu.edu.cn (M.L.); sunhj@.xmu.edu.cn (H.-J.S.); shuohuicao@xmu.edu.cn (S.-H.C.); chenz@xmu.edu.cn (Z.C.)

* Correspondence: zrni@xmu.edu.cn (Z.-R.N.)

SI. Skin effects of the hollow cylindrical conductive electrolyte

Based on the chamber structure in the electrochemical cell, an infinitely long hollow cylinder representing conductive electrolyte solutions, is assumed to be arranged coaxially in the z-axis direction and be immersed in an external applied alternating magnetic flux density B_1, which is linearly polarized along the y-axis direction (**Error! Reference source not found.**).

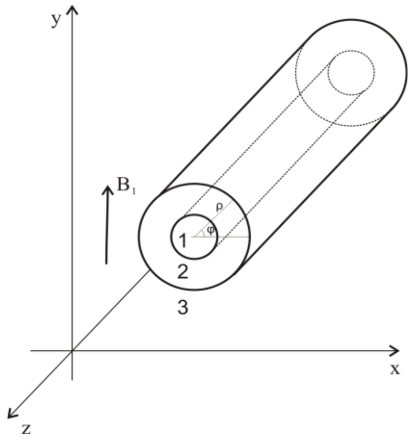

Figure S1. Cylindrical coordinate system used in the model of the electrolyte cell. The magnetic flux density B_1 is perpendicular to the cylinder axis. Region 1: non-conductive external standard; region 2: conductive electrolyte; region 3: air.

The penetration depth of currents induced by the time-varying magnetic field in the conductive cylinder depends on the electrolyte conductivity and the magnetic field frequency, given by

$$\delta = \sqrt{\frac{2}{\omega\mu\sigma}} \ . \qquad \text{(S.1)}$$

According to the NMR Lamour frequency of 500 MHz (wavelength λ of 600mm) and the conductivity 5 S/m of 0.1 M sulfuric acid solution, the penetration depth is found to be nearly 18 mm, which is only 2.6 times more than the diameter of a 5-mm-o.d. NMR tube as well as nearly equal to the length of the sample.

Consequently, interfaces of the tubular solution should not be treated simply as multi-layer planes, and the electromagnetic fields should be expressed in the following equations in a

cylindrical coordinates system,

$$\frac{\partial^2 E_z}{\partial \rho^2}+\frac{1}{\rho}\frac{\partial E_z}{\partial \rho}+\frac{1}{\rho^2}\frac{\partial^2 E_z}{\partial \varphi^2}+k^2 E_z=0\,, \tag{S.2}$$

$$H_\rho=\frac{i}{\mu_0\omega_0\rho}\frac{\partial E_z}{\partial \varphi}\,, \tag{S.3}$$

$$H_\varphi=-\frac{i}{\mu_0\omega_0}\frac{\partial E_z}{\partial \rho}\,, \tag{S.4}$$

where H_ρ and H_φ are the radial and azimuthal components of magnetic fields, E_z is the z-axis component of electric fields, and the complex wave number k is given by

$$k^2=\omega_0\mu_0\left(\omega_0\varepsilon\varepsilon_0-i\sigma\right). \tag{S.5}$$

Then magnetic fields H_ρ and H_φ are acquired by Equations (S.2) to (S.5) and transformed to spatial components H_x and H_y in Cartesian coordinates for being depicted conveniently. Figure S2 shows the spatial variation of the magnitude of these magnetic fields in the electrolyte with special conductivity and relative permittivity, indicating the nonuniform distribution of the fields.

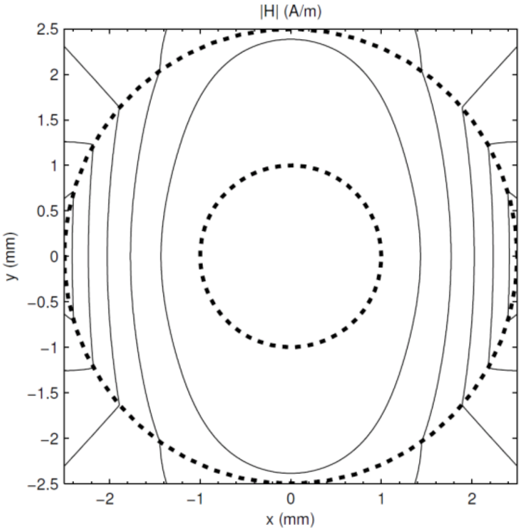

Figure S2. Spatial variation of the magnitude of RF B_1 in the electrolyte sample (ε_r = 78.5, σ = 20 S/m). The dash points represent outer and inner interfaces of the sample, whose diameters are 5 mm and 2 mm, respectively. Contour intervals represent the 0.5 percent of the value at the origin.

Phases of H_y along x-axis or y-axis shown in Figure S3 present that the increasing conductivity of electrolyte leads to the worsen phase distortion actually and the most serious disturbance is perpendicular to the y-axis, the direction of the linear polarizing field B_1.

Simultaneously, inflection points of both the amplitude and phase occur in the same direction at the outer boundary of the electrolyte model. The amplitude distortion of H_y results from perturbations caused by the conductive electrolyte, as shown in Figure S4 (a) (along x-axis) and (b) (along y-axis). These distortions are some less severe than those of phase. For instance, the phase drift of H_y across the x-axis exceeds 20° when the conductivity of electrolyte is greater than 40 S/m. At the same time, the variance of the amplitudes changes only about 5% of the magnetic field at the center. When the conductivity approaches zero, few phase drifts appear in both directions. However, due to the existence of the permittivity of the aqueous solution, the amplitude distortion still remains in a small range.

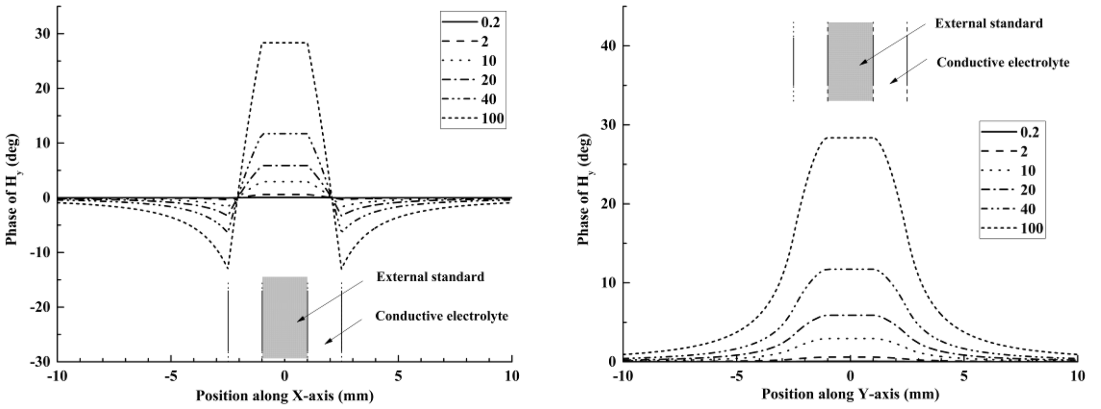

(a) (b)

Figure S3. Phase of H_y along **(a)** the X-axis or **(b)** the Y-axis in an electrolyte with different conductivity.

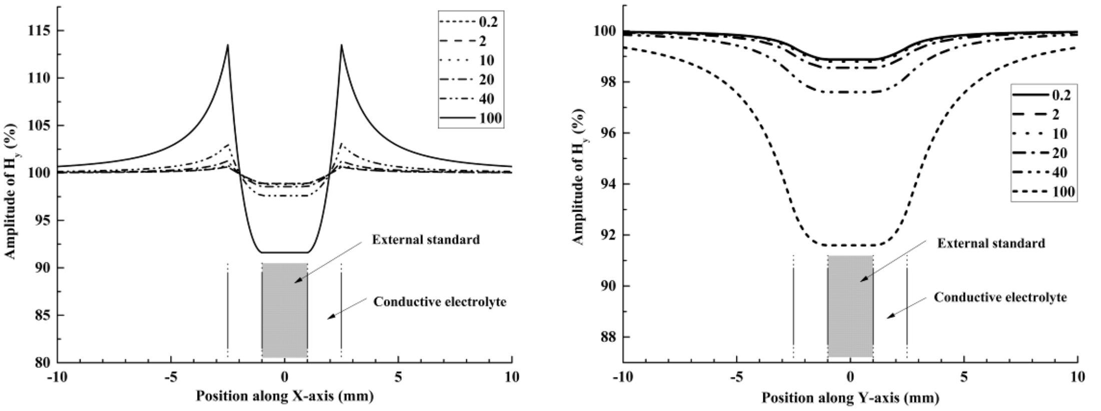

(a) (b)

Figure S4. Amplitude of H_y relative to the external applied magnetic field in electrolyte with different conductivity along **(a)** the X-axis or **(b)** the Y-axis.

Clearly, the distortion of the NMR RF field amplitude and phase definitely inclines to be nonlinearly exacerbated as the conductivity of the sample varies at any moment. Therefore, NMR signals of the resultant need to be calibrated specially to ensure high accuracy and precision in quantitative determination.

SII. Linear regressions of the NMR results versus gravimetric concentration

The linearity of the NMR response is verified using isopropanol solutions in D_2O (at ca. 0.10, 0.20, 0.30, 0.40, and 0.50 M), which are accurately gravimetrically prepared and admixed with 0.125 M sulfuric acid against 0.5 M ethylene glycol to be served as external standard. Figure S5 depicts linear regressions of the NMR results versus gravimetric concentration. More excellent linearity and relatively smaller intercept can be achieved by multiplying the constant K' to correct the intensity of NMR signal while the effect of conductive electrolytes is taken into account.

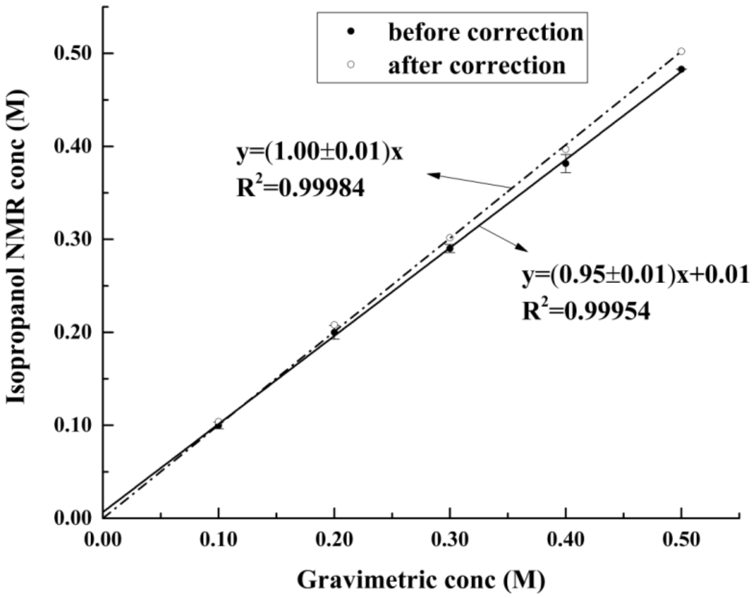

Figure S5. Concentration of isopropanol solutions determined by NMR against 0.5 M standard ethylene glycol solutions, vs gravimetric concentration of isopropanol: Linear regression before and after considering the effect of conductive electrolyte on the intensity of NMR signal.
